# Laparoscopic resection of a retroperitoneal schwannoma located in the hepatic hilus

**DOI:** 10.1186/s40792-015-0024-6

**Published:** 2015-02-19

**Authors:** Tetsuro Maruyama, Yuji Sugamoto, Daisuke Miyagishima, Toru Fukunaga, Kentaro Tasaki, Nobuyoshi Takeshita, Tomohide Tamachi, Yo Asai, Takashi Hosokawa, Eiichiro Ninomiya, Masayuki Kimura

**Affiliations:** Department of Surgery, Numazu City Hospital, Shizuoka, Japan; Department of Gastroenterology, Numazu City Hospital, Shizuoka, Japan; Department of Pediatric Surgery, Numazu City Hospital, Shizuoka, Japan; Numazu City Hospital, Aza Haru no Ki 550, Higashi Shiiji, Numazu shi, Shizuoka Japan

**Keywords:** Retroperitoneal schwannoma, Laparoscopic resection, Preoperative diagnosis

## Abstract

Schwannomas are tumors and commonly occur in the head and neck region; however, they rarely present in the retroperitoneum. A 79-year-old man was admitted to our hospital for a follow-up of a tumor in the hepatic hilus. A 2.8 × 2.5 cm solid tumor located between the hepatic hilus and common hepatic artery was originally identified, and the size of the tumor had increased from 2.0 × 2.0 cm to 2.8 × 2.5 cm over the course of 3 years. The patient underwent percutaneous sonopsy, and the tumor was subsequently diagnosed as a benign schwannoma. Since the patient wished to undergo an operation, we performed laparoscopic surgery. During the operation, the tumor was detected in the retroperitoneal space, where it was strongly adhered between the left gastric artery and common hepatic artery. At this point, no major vessels had vascularized the tumor. We then completely removed the tumor from the retroperitoneal space without any complications. The clinical course was uneventful, and the patient was discharged on postoperative day 4 without any symptoms. Later, a definitive histopathologic examination revealed a benign schwannoma. Here, we report this rare case of a retroperitoneal schwannoma located in the hepatic hilus.

## Background

Schwannomas are tumors arising from Schwann cells of the peripheral nerves. Although most schwannomas occur in the cephalocervical region and limbs, tumors in the retroperitoneal location are rare. Retroperitoneal tumors have traditionally been excised using a standard open technique. With recent advances in the field of minimally invasive surgery, 18 cases of retroperitoneal schwannomas excised by laparoscopic approaches have been reported, including our case. Similar to our case, there was only one other case where the tumor was located behind the lesser omental sac, and there were no cases diagnosed before the operation. Typically, it is very difficult to diagnose retroperitoneal tumors before the operation, since both clinical and radiologic features specific to schwannoma are usually absent. Here, we report of a rare case of retroperitoneal schwannoma, which was successfully diagnosed preoperatively and completely excised by laparoscopic approaches.

## Case presentation

**Figure 1:**
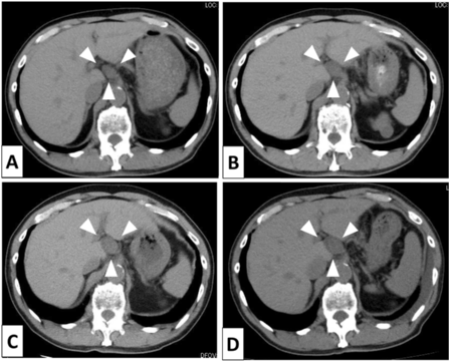
**The CT scans of the past 3 years. (A)** March, 2011. **(B)** March, 2012. **(C)** April, 2013. **(D)** January, 2014.

**Figure 2:**
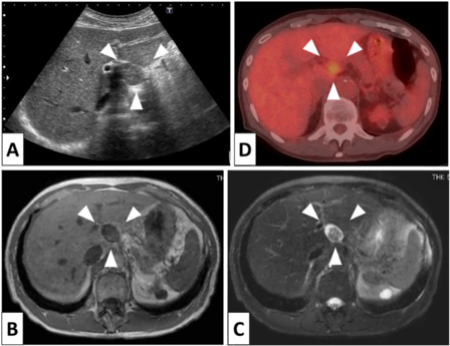
**The results of Abdominal ultrasonography and weighted images of MRI. (A)** Abdominal ultrasonography revealed a 2.8 × 2.5 cm solid tumor between hepatic hilus and common hepatic artery. **(B)** T1-weighted image of MRI showed a heterogenous low-intensity lesion. **(C)** T2-weighted image of MRI showed a heterogenous high-intensity lesion except for the central area. **(D)** PET/CT showed a low FDG-uptake mass (max SUV = 3.6).

A 79-year-old man with chronic renal failure, atrial fibrillation, abdominal aortic aneurysm, and a tumor of the hepatic hilus was being followed up for a period of 3 years at his local hospital. He was later admitted to our hospital and had no family history. His general physical and abdominal examinations revealed normal findings. The results of laboratory investigations showed slight anemia (hemoglobin concentration, 12.0 g/dl) and renal failure (serum creatinine concentration, 1.87 mg/dl), while the results of the other routine biochemical profile parameters were within normal limits. Computed tomography (CT) revealed a 2.8 × 2.5 cm solid tumor between the hepatic hilus and common hepatic artery (CHA) (Figure 1D). Next, we requested the results of his CT scans for the past 3 years from his local hospital. When viewed as a time series, the CT scans revealed an increase in tumor size from 2.0 × 2.0 to 2.8 × 2.5 cm over a span of 3 years (Figure 1). Because there was a possibility of malignancy of the tumor, we made a close examination by patient’s request. Abdominal ultrasonography revealed a solid and round tumor between the hepatic hilus (Figure 2A). Magnetic resonance imaging (MRI) showed a heterogeneous low-intensity lesion on a T1-weighted image and a heterogeneous high-intensity lesion on a T2-weighted image, except for the central area (Figure 2B,C). A low fluorodeoxyglucose (FDG)-uptake mass (max standardized uptake value [SUV] = 3.6) was observed on positron emission tomography and computed tomography (PET/CT) whole body scan using F-18 FDG (Figure 2D). Temporal change in the tumor size suggested the malignant nature of the tumor, although it was considered a benign schwannoma by these radiologic findings. As the possibility of malignancy could not be denied, the patient underwent percutaneous transhepatic sonopsy. Microscopic examination revealed fascicles of benign-appearing spindle cells that were positive for S-100. Ultimately, we diagnosed the tumor as a benign schwannoma and suggested follow-up with examination to a patient. But the patient strongly wished to undergo an operation; therefore, we performed laparoscopic surgery.

**Figure 3:**
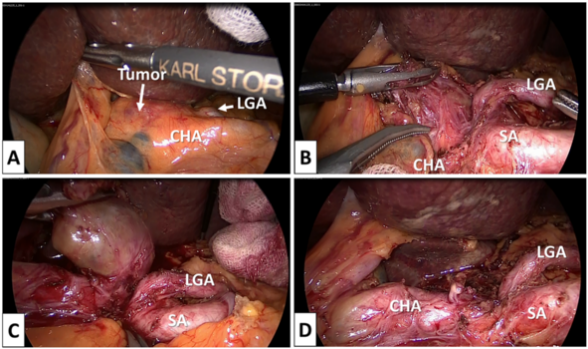
**Laparoscopic view during surgery. (A)** The tumor was detected in the retroperitoneal space between LGA and CHA. **(B)** The tumor was strongly adhered to LGA and CHA. **(C)** We could observe network of peripheral nerve around the CHA entering the tumor. **(D)** After resection of the tumor from retroperitoneal space. LGA, left gastric artery; CHA, common hepatic artery; SA, splenic artery.

**Figure 4:**
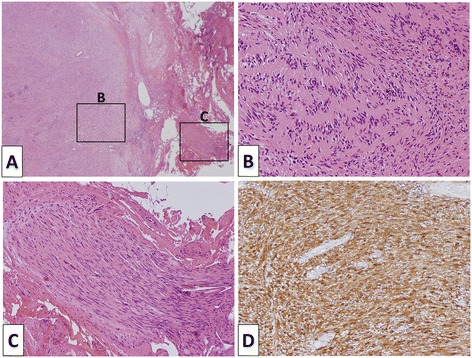
**Microscopic findings. (A)** A fascicular arrangement of strand spindle cells. **(B)** Palisading pattern. **(C)** A normal peripheral nerve entering into the tumor. **(D)** Immunohistochemical stains were positive for S-100.

Under general anesthesia, the patient was placed in the supine position with his legs separated. The primary surgeon and scrub nurse stood to the right of the patient, an assistant surgeon equipped with a camera stood between the patient’s legs, and another assistant surgeon stood to the left of the patient. A primary 12-mm trocar was inserted into a trans-umbilical incision to introduce a laparoscope. A second 12-mm trocar was placed at the right upper mid-inguinal vertical line. Another three 5-mm trocars were placed at the right subcostal line, the left subcostal line, and left upper mid-inguinal vertical line, similar to the placements for laparoscopic gastric resection in our hospital. A monitor, CO_2_ pneumoperitoneum (10 mmHg), a laparoscope (EndoCAMeleon, KARL STORZ, Tuttlingen, Germany), an ultrasonic dissector (SonoSurg, Olympus, Tokyo, Japan), and routine laparoscopic instruments were used. The abdomen was explored, and there was no evidence of intraperitoneal and hepatic metastases. The liver was pulled up from the subxiphoid using a 3-mm forceps, and the stomach was pulled down by the assistant surgeon. The tumor could be observed through the lesser omental sac; therefore, the lesser omental sac was opened. The tumor was detected in the retroperitoneal space between the left gastric artery (LGA) and CHA (Figure 3A). After opening the peritoneal membrane over the tumor, a smooth yellow tumor was visible and the tumor was strongly adhered to the LGA and CHA (Figure 3B). The tumor was carefully dissected from the surrounding tissue, and a surrounding major vessel was preserved. We found a network of peripheral nerves around the CHA entering the tumor, and we dissected them one by one (Figure 3C). No major vessels had entered the tumor. We removed the tumor completely from the retroperitoneal space and excised it through the umbilical incision using a surgical globe (Figure 3D). A drain was inserted into the retroperitoneal space between the LGA and CHA. The duration of the operation was 200 minutes, and blood loss was about 5 g. The patient started oral intake and ambulation on the first postoperative day, and the drain was withdrawn on the second postoperative day. The clinical course was uneventful, and the patient was discharged on postoperative day 4 without any symptoms. The resected tumor was measured 30 × 21 × 18 mm. On pathological examination, the tumor was encased in a surrounding capsule and there was no exposure of a tumor. A fascicular arrangement of strand spindle cells with a palisading pattern and a normal peripheral nerve entering into the tumor was observed (Figure 4A,B,C). The area of Antoni-A was larger than the area of Antoni-B in the tumor. There was no definitive evidence of nuclear abnormalities in the tumor. On immunohistochemistry, the tumor was positive for S-100 (Figure 4D), and a definitive histopathologic examination revealed a benign schwannoma.

## Discussion

Schwannomas are neuroectodermal tumors arising from Schwann cells of the nerve sheaths, and they most commonly occur in the head and neck region (44.8% of cases), upper limbs (19.1%), and lower limbs (13.5%). In contrast, it is relatively rare in the retroperitoneum, with an occurrence rate of 0.7% [1]. In the retroperitoneal space, schwannomas occur most commonly in patients between 20 and 50 years of age, being slightly more common in women [2].

If a schwannoma is located in the retroperitoneal space, it grows without symptoms. If it is considerably large, various symptoms can be present, but there may be no specific clinical condition [3]. In other words, most retroperitoneal tumors are asymptomatic and are diagnosed incidentally, while evaluating the patient for some other disease.

There is no gold standard diagnostic method for retroperitoneal schwannoma; therefore, it is difficult to provide a definitive diagnosis of retroperitoneal schwannoma before the operation. An ordinary schwannoma is depicted as a well-defined and inhomogeneous low-density mass on CT images [4]. On MRI, schwannomas are seen as masses with hypointensity on T1-weighted images and hyperintensity on T2-weighted images [5]. Schwannomas typically appear as solitary, well-encapsulated masses, which are firm and round with a smooth surface. Although these findings are not typical imaging features, they are helpful in treatment planning because they provide information on the invasion of other structures [6]. Sonopsy is a useful technique to diagnose a retroperitoneal tumor such as retroperitoneal schwannoma. Kudo et al. said that endoscopic ultrasound-guided fine-needle aspiration is useful to diagnose retroperitoneal schwannoma [7]. But, it is difficult to find a safe route from the skin to the tumor, especially in dediastinal, perirectal, or retroperitoneal masses [8]. In our case, it was possible to perform percutaneous transhepatic approach to the tumor and diagnose preoperatively.

On the other hand, the potential for seeding of malignant cells by sonopsy techniques is a cause for concern. Daneshmand et al. said that sonopsy is not recommended in schwannoma [9]. But there are no reports of dissemination of schwannoma caused by sonopsy. In our case, we thought that sonopsy is necessary to diagnose a retroperitoneal tumor because temporal change in the tumor size suggested the malignant nature of the tumor. If doing sonopsy for retroperitoneal tumor, it should be done after doing enough informed consent.

A definitive diagnosis is based on pathological, histological, and immunohistochemical findings. Histologically, schwannomas consist of compact cellular lesions (Antoni type A tissue) and loose, hypocellular myxoid lesions with microcystic spaces (Antoni type B tissue). Additionally, almost all schwannomas show intense immunohistochemical staining for S-100 protein, confirming the neuroectodermal origin of the tumor cells [10].

The malignancy rate of schwannomas range from 1.7 to 30.7%, and the relapse rate is as high as 61% [3]. Even in pathologically benign cases, relapse is possible, with the relapse rate reported as 4.3%, and the malignant transformation rate, as 12% [11]. According to Nakashima et al., a tumor size larger than 5.5 cm, symptoms, the absence of calcifications, irregular margins, and cystic degeneration or necrosis may all be predictors of primary retroperitoneal malignant tumors [12].

According to Qiang et al. [13], the ideal treatment for schwannoma is complete resection of the tumor and capsule without injuring the adherent organs. In our case, the tumor of the patient was diagnosed as benign schwannoma, and the patient had a lot of complications. Maybe the patient did not need tumor resection. But the patient strongly wished to undergo an operation because of the possibility of malignancy. We performed laparoscopic surgery after enough informed consent with the patient and his family.

Since the first laparoscopic retroperitoneal resection of schwannoma in 1996, 18 cases have been reported including our case (Table 1) [3,6,14-28]. Based on the available data, the average size of tumors, operation time, intraoperative blood loss, and postoperative length of stay in the hospital are 52 mm (range, 19 to 83 mm) in maximum diameter, 180 minutes (range, 95 to 306 min), 91 ml (range, 5 to 200 ml), and 6.3 days (range, 1 to 14 days), respectively. There were no severe operative or postoperative complications in almost all cases. Endoscopic surgery, which enables detailed observation with minimal invasiveness and magnified vision, has become a viable option for this procedure. In our case, we found a network of peripheral nerves around the CHA, entering the tumor, and we dissected them individually.

**Table 1:** **Eighteen cases of retroperitoneal schwannoma resected by laparoscopic surgery**

**Author**	**Year**	**Sex**	**Age**	**Location**	**Size (mm)**	**Operative time (min)**	**Blood loss (ml)**	**Complication**	**Postoperative day**	**Diagnosis**
Melvin [14]	1996	F	36	Presacral space	Not described	Not described	Not described	None	1	Not described
Ohigashi [15]	1999	F	28	Between the right kidney and IVC	29 × 27 × 25	180	25	None	Not described	Benign schwannoma
Nishio [16]	1999	F	41	Anterior to the right psoas muscle	50 × 50	195	200	None	14	Benign schwannoma
Descazeaud [17]	2003	F	62	Retroperitoneal	80 × 50	Not described	Not described	Not described	Not described	Benign schwannoma
Funamizu [18]	2004	M	55	Behind the lesser omental sac	60 × 45 × 40	230	15	None	10	Benign schwannoma
Morrison [19]	2004	F	62	Between the left adrenal gland and kidney	72 × 47 × 42	105	Minimal	None	1	Benign schwannoma
Singh [20]	2005	F	62	Between the right kidney and vertebral column	20 × 10	Not described	Not described	Not described	Not described	Benign schwannoma
Inokuchi [21]	2006	F	35	Right adrenal gland	75 × 65 × 25	Not described	Not described	None	11	Benign schwannoma
Park [22]	2007	F	44	On the left obturator nerve	19 × 18 × 18	Not described	Not described	None	4	Benign schwannoma
Kang [23]	2008	F	59	Between IVC and pancreaticoduodenal unit	53	230	150	None	3	Schwannoma
Yoshino [6]	2008	M	67	Left adrenal gland	83 × 65 × 45	Not described	Not described	None	10	Benign schwannoma
Rao [24]	2009	M	42	Behind the pancreas	30 × 27	210	100	None	6	Schwannoma
Gorgun [25]	2010	F	33	Between the right kidney and IVC	50 × 40	120	200	none	5	Benign schwannoma
Sasaki [26]	2010	F	69	Anterior to the left psoas muscle	55	95	15	None	11	Benign schwannoma
Mahesh [27]	2010	F	41	Between the aorta and kidney	80 × 80	140	100	None	3	Schwannoma
Asakage [3]	2012	F	34	Dorsal side of the AVC	55 × 35	306	100	None	7	Benign schwannoma
Okuyama [28]	2014	F	54	Right side of the pelvic cavity	50 × 40	150	Minimal	None	4	Benign schwannoma
Our case	2014	M	79	Behind the lesser omental sac	30 × 21 × 18	200	5	None	4	Benign schwannoma

## Conclusion

Retroperitoneal schwannomas are generally benign, localized, hypovascular, and noninvasive and have a considerable amount of adherent fibers, which makes retraction less difficult during the laparoscopic procedure. Laparoscopic surgery is very useful and feasible in the diagnosis and treatment of retroperitoneal schwannoma, associated with minimal invasiveness and early postoperative recovery. Even in pathologically benign cases, relapse is possible; hence, careful follow-up is required.

## Consent

Written informed consent was obtained from the patient for the publication of this case report and any accompanying images. A copy of the written consent is available for review by the Editor-in-Chief of this journal.
